# Postnatal care by provider type and neonatal death in sub-Saharan Africa: a multilevel analysis

**DOI:** 10.1186/1471-2458-14-941

**Published:** 2014-09-10

**Authors:** Kavita Singh, Paul Brodish, Erica Haney

**Affiliations:** Department of Maternal and Child Health, Gillings School of Global Public Health, University of North Carolina at Chapel Hill, Chapel Hill, NC USA; Carolina Population Center, University of North Carolina at Chapel Hill, Chapel Hill, NC USA; Department of Public Policy, University of North Carolina at Chapel Hill, Chapel Hill, NC USA

**Keywords:** Infant mortality, Neonatal mortality, Skilled providers, Unskilled providers, Scale-up

## Abstract

**Background:**

Globally postnatal care (PNC) of the newborn is being promoted as a strategy to reduce neonatal deaths, yet few studies have looked at associations between early PNC and neonatal outcomes in sub-Saharan Africa. In this study we look at the associations of PNC provided on day 1 and by day 7 of life by type of provider – skilled (doctor, midwife or nurse or unskilled (traditional birth attendant or community health worker) on neonatal death on days 2 to 7 and days 2 to 28.

**Methods:**

Data from 10 African countries with recent (from 2009 onwards) Demographic and Health Surveys are pooled and used in a multilevel logistic regression analysis to study associations between the PNC variables with the mortality outcomes after controlling for relevant socioeconomic and maternal factors (including antenatal care, skilled delivery, tetanus immunization and ever breastfed).

**Results:**

Findings indicate that PNC, whether provided by a skilled or unskilled provider, is protective against both neonatal death outcomes. Unskilled PNC on day 1was associated with a 32% decrease in the probability of death (compared to no PNC on day 1) during days 2 to 28 after controlling for other factors (OR: 0.68; 95% CI: 0.48, 0.97). Both skilled and unskilled PNC by day 7 were associated with reduced neonatal death during days 2 to 7 (Skilled: OR: 0.40; 95% CI 0.18, 0.88; Unskilled: OR 0.34; 95% CI 0.23, 0.52) and days 2 to 28 (Skilled: OR: 0.51; 95% CI 0.35, 0.75; Unskilled: OR 0.34; 95% CI 0.30, 0.38). There were also significant associations between four or more antenatal care visits and ever breastfed with both outcomes.

**Conclusion:**

PNC is an important strategy to reduce neonatal death. While postnatal care by a skilled provider is a preferred strategy, PNC provided by unskilled providers can also serve as an intermediate implementation approach as countries strive to reach more newborns and save more lives.

## Background

The neonatal period, the first 28 days of life, represents a particularly vulnerable period in an individual’s life with the latest estimates indicating that 2.76 million deaths occur during this short time period [1]. Neonatal deaths actually account for 40% of under-five deaths [2, 3]. A combination of factors is responsible for these deaths including limited health care access, delayed recognition of complications, delayed care seeking and inadequate antenatal, intrapartum and postnatal care [4, 5]. Despite the high burden of neonatal mortality, Africa has seen the slowest improvements in neonatal mortality rates with a decline of only 19% from 1990 to 2010 in contrast to the 43% decline witnessed in high income countries [6]. With only 15.5% of the world’s population [7], Africa accounts for 39% of neonatal deaths with the majority of these deaths occurring specifically in sub-Saharan Africa [6].

A large number of early neonatal deaths could be averted through implementation of simple and low cost interventions [8]. Some of these interventions are part of care that can be provided during the postnatal period, which is defined as the first six weeks after delivery. This is a critical period for both the mother and infant in that the majority of maternal deaths and a high proportion of infant deaths occur during this time. Early postnatal care (PNC) interventions that can be provided to newborns include pneumonia case management and referral, promotion of breastfeeding, prevention and management of hypothermia and kangaroo care (for low birth weight babies) [8–10]. The interventions and promotion of health behaviours during PNC check-ups could be an effective means to prevent some of the main causes of neonatal death, particularly some of the deaths to pre-term newborns and deaths due to sepsis, meningitis, pneumonia and diarrhea. Intrapartum-related birth asphyxia is a complication that must be addressed during labor, delivery and immediately afterwards, thus PNC may not lead specifically address this cause of neonatal mortality. However follow-up care for these infants is important, particularly if they experience long-term complications, and PNC can play a role in connecting caregivers to available services if and when such services exist.

The majority of neonatal deaths occurring after 48 hours of life can be prevented by care provided immediately after delivery [11]. The World Health Organization recommends that the first PNC check-up between health provider and newborn be within the first hour of life, if the delivery is within a facility, and within the first 24 hours, if delivery is at home. Follow-up PNC should occur within two to three days, six to seven days and at six weeks. The content of PNC should include education regarding immediate and exclusive breastfeeding, hand washing, warming of the baby, hygienic cord cleaning, as well as examination of danger signs for the mother and baby and appropriate referral for care [11, 12]. Additional contact is recommended for high-risk cases such as newborns who are low birth weight and/or pre-term and babies born to HIV-infected mothers.

Only a few studies in sub-Saharan Africa have looked at the effects of PNC on newborn outcomes. A study in Zambia found that midwives who provided consistent home-based PNC at three, seven, 28, and 42 days after delivery improved women’s ability to identify risk among newborns and utilize health services more frequently than women who received fewer PNC visits [13]. The more recent Newhints trial in Ghana also used a home-based approach in its focus on providing both antenatal care (ANC) and PNC for newborns. Though neonatal mortality was not significantly different between the intervention and comparison areas, coverage of key PNC interventions was significantly higher in the intervention areas [14]. Other studies have shown that PNC provided by unskilled providers, particularly traditional birth attendants (TBAs), have been effective in improving newborn outcomes [15]. Both the studies in Zambia and Ghana and additional studies of TBAs have used home-based PNC visits [13–15] which may be a contextually appropriate, locally adaptive approach for many countries given that only 46% of women in sub-Saharan Africa delivered their last child in a health facility [16]. In 2009 WHO and UNICEF issued a joint statement advocating home PNC visits for newborn babies if their caregivers were unable to bring them to a health facility [11].

The objective of this study is to examine associations between PNC early checks (on day 1 and within the first 7 days of life) on neonatal death in sub-Saharan Africa using a large and recent sample of data from 10 countries. In our analysis we categorize the PNC variables to study whether PNC provided by an unskilled birth attendant such as a TBA or community health worker is as effective as care provided by a skilled birth attendant (SBA), defined as a doctor, midwife or nurse. PNC provided by a SBA may not yet be a feasible option for many countries in Africa due to shortages of SBAs, particularly in rural areas, thus the programmatic relevance of understanding differentials by provider type.

## Methods

### Data and sample

Demographic and Health Survey (DHS) data were pooled from ten countries in sub-Saharan Africa - Burkina Faso, Burundi, Cameroon, Ethiopia, Malawi, Rwanda, Senegal, Tanzania, Uganda, and Zimbabwe [17]. These countries were selected because they had the most recent (2009 or later) data available. DHS surveys are nationally representative population-based surveys with large sample sizes and in all randomly selected households, women age 15-49 are eligible to participate. The sample is usually based on a stratified two-stage cluster design. The first stage is the sample enumeration area (SEA) or cluster, generally drawn from Census files. In the second stage, within each SEA, a sample of households is selected from a list of households. The sample is generally representative at the national level, residence (urban-rural), and regional (departments, states) levels. Women who had not given birth within five years prior to the survey were excluded from the analysis because of the focus on assessing recent neonatal mortality. For newborns who died on the first day of life, the DHS does not record whether they died within minutes of birth (before they would be eligible for a PNC check-up) or after one hour when they could have been eligible if they were delivered in a facility. Because of this potential for left censoring of the data, only women whose babies survived the first day of life were included in the analysis. The sample consisted of 31,799 women across the ten countries.

This analysis was reviewed by the Institutional Review Board at the University of North Carolina-Chapel Hill and exempted from needing approval. The analysis is purely secondary and no identifying information was used or available.

### Outcome variables

There were two outcome variables – neonatal death between days 2 to 7 and neonatal death between days 2 and 28. Newborns who were less one month would have an unknown survival status in the first 28 days of life. In order to correct for this right censoring, all neonates who were less than one month old were not included in the analysis for the neonatal death on days 2 to 28 outcome. We were not able to account for right censoring for the outcome on neonatal death between days 2 and 7 because only month and year of birth, and not day, are recorded in the DHS. This should not affect the results for our neonatal death on day 2 to 7 analysis because of small numbers of newborns less than 7 days old.

### Key independent variables

The PNC independent variables were 1) having a first PNC check on the first day of life and 2) having a first PNC check within the first seven days of life. These two variables were not mutually exclusive and were included in separate regression analyses. The coding categories for each of these independent variables were 1) yes, checked by a SBA (doctor, midwife or nurse), 2) yes, checked by an unskilled birth attendant (TBA or community health worker) and 3) not checked.

In addition to the PNC variables, we studied available maternal health variables relevant to our two outcomes. The maternal health independent variables were receipt of tetanus toxoid before or during pregnancy, four or more antenatal (ANC) visits, whether the newborn was ever breastfed, and the skill of the birth attendant and location of delivery (unskilled at home, unskilled in a facility, skilled at home, and skilled in a facility). Unskilled delivery in a facility is uncommon but may happen in under-staffed or crowded facilities. Information on birth weight and gestational age were not available, and thus could not be included in the analysis.

### Control variables

The socio-demographic control variables were maternal age in years (15-19, 20-24, 25-34, 35-49), parity (one, two to three, and four or more), education (none, primary, secondary or greater), urban or rural residence and wealth quintile (poorest, second poorest, middle, second richest, richest). The DHS wealth index is constructed based on household ownership of assets, housing material and types of access to water and sanitation. The sociodemographic independent variables were chosen based on the literature and all were included in the regression models as control variables.

### Analysis

Because the data were clustered within countries, the standard assumption of logistic regression that individual respondents are independent across countries and that there is equal variance across countries did not hold. A multilevel regression framework was used to account for this clustered data structure. Based on modelling recommendations, a null or base model was run including only the dependent variable neonatal death on days 2 to 7 or neonatal deaths on days 2 to 28 to establish the degree of variance at the country level, in order to validate use of a multilevel framework. Next, one of the PNC variables was added to test for significance of the predictor variable. Finally, all the individual-level demographic control variables were added to the model. All models were estimated with the gllamm command in Stata MP version 12.1 and utilized DHS-specific sampling weights for each country.

## Results

### Sample characteristics

**Figure 1:**
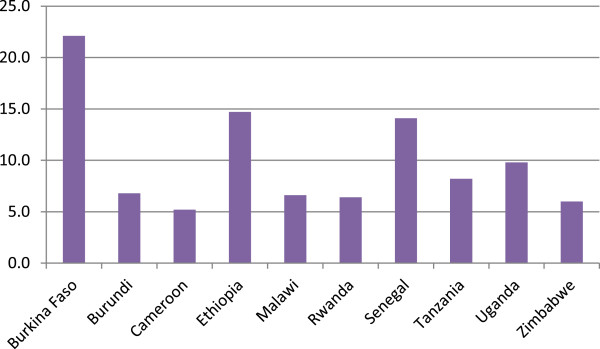
**Distribution of the weighted sample by country.**

Table 1 summarizes the pooled survey sample characteristics. Just over one third of respondents reported having the recommended four or more ANC visits, and approximately 87% reported receiving tetanus toxoid either before or during the most recent pregnancy. Overall, 56% of deliveries were performed by an unskilled birth attendant at home and 41% by a SBA in a facility, with less than two percent being performed by an unskilled attendant in a facility or a SBA at home. Only 16% of women reported a PNC check-up for their child on the first day of birth with six percent reporting it was given by an unskilled provider and 10% reporting provision by a SBA. Nearly 32% of women reported a PNC check-up within the first week, 12% by an unskilled provider and 19% by a SBA. Over 98% of women reported ever breastfeeding the child. Forty-six percent of respondents were aged 25-34, and 85% were multiparous. Figure 1 presents the distribution of the total sample by country.

**Table 1:** **Sample characteristics: survey-weighted percentages (n = 31,799)**

Characteristic	Percentage
Antenatal	
Four or more ANC visits	37.8
Tetanus toxoid before or during pregnancy	86.8
Delivery	
Unskilled at home	56.1
Unskilled in a facility	1.5
Skilled at home	1.5
Skilled in a facility	40.9
Postnatal	
Visit on day 1	
None	83.9
Unskilled	5.7
Skilled	10.4
Visit by day 7	
None	68.2
Unskilled	12.4
Skilled	19.3
Ever breast fed	98.6
Demographic	
Age (years)	
15–19	4.3
20–24	20.2
25–34	46.3
35–49	29.2
Parity	
1	16.4
2–3	32.2
4+	51.4
Education	
None	52.6
Primary	36.2
Secondary+	11.2
Residence	
Urban	18.9
Wealth quintile	
Lowest	23.7
Second	22.4
Middle	20.8
Fourth	18.9
Highest	14.3

NOTE: Skilled Birth Attendant (SBA): doctor, nurse or midwife; Unskilled: traditional birth attendant or community health worker.

### Neonatal death by PNC

Table 2 shows that overall there were approximately 8.8 neonatal deaths per 1,000 births (or approximately 280 deaths) during days 2 to 7 among those receiving no PNC on day 1, with fewer deaths occurring among those receiving unskilled (6.6 per 1000) or skilled (6.8 per 1000) PNC on day 1. For the outcome of deaths during days 2 to 28 among those with no PNC on day 1 the rate increased to 14.2 per 1,000 (or approximately 450 deaths) compared to 8.7 per 1000 for unskilled PNC and 10.6 per 1000 for skilled PNC. When studying PNC by day 7, the results followed a similar pattern with the highest death rates among newborns not receiving any PNC, followed by newborns receiving skilled PNC and then newborns receiving unskilled PNC.

**Table 2:** **Survey-weighted neonatal death (per 1,000), by timing and type of postnatal care (n = 31,799)**

		Death rate per 1,000	
		Days 2 to 7	Days 2 to 28
PNC on day 1	None	8.8	14.2
	Unskilled	6.6	8.7
	Skilled	6.8	10.6
PNC by day 7	None	10.3	16.2
	Unskilled	4.1	6.6
	Skilled	4.6	8.4

### Multilevel modelling results

Table 3 shows the multilevel logistic modelling results for the two outcomes. The significance of the random effect indicates that the intercepts for the regression model are significantly different across the countries, and therefore a multi-level analysis in which clustering by country is accounted for is appropriate. Compared to neonates receiving no PNC on day 1, unskilled PNC was associated with a 32% decrease in the probability of death during days 2 to 28 of life after controlling for other factors (OR 0.68; 95% CI 0.48, 0.97). Compared to neonates receiving no PNC by day 7, unskilled PNC was significantly associated with a 66% decrease in the probability of death during days 2 to 7 (OR 0.34; 95% CI 0.23, 0.52) and days 2 to 28 (OR 0.34; 95% CI 0.30, 0.38) controlling for other factors Skilled PNC by day 7 was significantly associated with a 60% decrease in the probability of death during days 2 to 7 (OR 0.40; 95% CI 0.18, 0.88) and a 49% decrease in the probability of death during days 2 to 28 (OR 0.51; 95% CI 0.35, 0.75), controlling for all other factors. As expected, there was a significant protective effect for four or more antenatal visits in all models, and a strongly protective effect for ever having breastfed. Tetanus immunization and unskilled delivery in a facility (compared to unskilled delivery at home) were significantly, however inconsistently, associated with a decrease in death rates for some of the models.

**Table 3:** **Parameter estimates (odds ratio) for multilevel weighted logistic regression models of death on days 2 to 7 and death on days 2 to 28 by PNC type, maternal health factors and socio-demographic controls (n = 31,799)** ^**a**^

	Deaths in the first week						Deaths in the first month					
Explanatory variables	OR	(95% CI)		OR	(95% CI)		OR	(95% CI)		OR	(95% CI)	
Fixed effects												
Post natal care												
Visit on first day												
None	1.00						1.00					
Unskilled	0.87	(0.55, 1.38)					0.68	(0.48, 0.97)	*			
Skilled	0.77	(0.43, 1.38)					0.79	(0.55, 1.15)				
Visit within first week												
None				1.00						1.00		
Unskilled				0.34	(0.23, 0.52)	***				0.34	(0.30, 0.38)	***
Skilled				0.40	(0.18, 0.88)	*				0.51	(0.35, 0.75)	***
Ever breastfed												
No	1.00			1.00			1.00			1.00		
Yes	0.02	(0.01, 0.04)	***	0.02	(0.01, 0.04)	***	0.03	(0.02, 0.04)	***	0.03	(0.02, 0.04)	***
Delivery characteristics												
Unskilled at home	1.00			1.00			1.00			1.00		
Unskilled in a facility	0.21	(0.10, 0.41)	***	0.28	(0.12, 0.66)	**	0.68	(0.36, 1.29)		0.85	(0.48, 1.50)	
Skilled at home	0.37	(0.09, 1.53)		0.39	(0.10, 1.54)		0.69	(0.35, 1.36)		0.72	(0.39, 1.35)	
Skilled in a facility	1.20	(0.68, 2.13)		1.39	(0.76, 2.54)		0.95	(0.67, 1.35)		1.05	(0.72, 1.52)	
Antenatal care												
Tetanus toxoid before/during pregnancy												
No	1.00			1.00			1.00			1.00		
Yes	0.66	(0.45, 0.95)	*	0.71	(0.48, 1.04)		0.63	(0.46, 0.88)	**	0.67	(0.49, 0.93)	*
Antenatal visits												
< 4	1.00			1.00			1.00			1.00		
≥ 4	0.58	(0.43, 0.78)	***	0.59	(0.44, 0.79)	***	0.58	(0.45, 0.74)	***	0.58	(0.45, 0.76)	***
Random effects												
Country-level variance (SE)^b^	0.80	(0.67, 0.95)	*	0.74	(0.63, 0.88)	***	0.74	(0.61, 0.89)	**	0.70	(0.59, 0.82)	***
Log-likelihood		-1306.96			-1293.25			-1985.77			-1969.57	
AIC		2631.91			2604.50			3991.53			3959.14	
Log-likelihood ratio test (Chi-square)^c^		498.20	***		490.10	***		580.31	***		569.25	***

Parameters for predictors (fixed effects) are reported as odds ratio; for random effects, the parameter is the variance.

*p < 0.05; **p < 0.01, ***p < 0.001.

^a^Full model including all demographic control variables: age, parity, education, residence (urban/rural), and wealth quintile.

^b^Significance of random effects evaluated by comparing model with a similar one in which random effects have been constrained to be zero.

^c^Compared to null model with no covariate.

## Discussion

PNC is being promoted as a strategy to save newborn lives, but few studies have evaluated associations between PNC and newborn outcomes in sub-Saharan Africa [13–15]. This study is among the first to evaluate associations between PNC by provider type and neonatal death. Overall findings from this study indicate that countries should continue to promote PNC as a means to prevent neonatal mortality and improve newborn health.

Results from our analysis indicate that PNC is associated with saved lives. This finding could be due to improved health from interventions provided and knowledge shared between providers and caregivers regarding newborn care. ANC and tetanus immunization were also consistently protective against both neonatal death outcomes. These services are essential elements in a continuum of care approach for mothers and newborns [18].

Our results suggest that PNC whether provided by either a skilled or unskilled health worker, is better than no PNC in terms of saving the lives of newborns. However, results from our unadjusted analysis and also the regression analyses indicated that unskilled PNC appears more effective than skilled PNC. This somewhat unexpected finding may be due to the fact that women with pregnancy and/or delivery-related complications may be more likely to come to a facility (than women without complications) and may be presenting late. A similar finding has been documented in another study looking at the effect of delivery with a skilled birth attendant on neonatal mortality [19]. Quality of care provided at the facility may be another issue related to this finding [19–21] and highlights the importance of training health workers to handle newborn complications. In many settings SBAs have not been given specific training or supplies to prevent deaths due to neonatal complications. Also in our study we had to exclude deaths on the very first day of life. Many of these deaths are due to intrapartum-related birth asphyxia or prematurity [2], and a properly trained and equipped SBA could prevent many such deaths. In contrast an unskilled birth attendant would be unlikely to have the skills or equipment to prevent many of the deaths on day 1. Thus PNC with a SBA is the ideal approach, while PNC with an unskilled provider should be considered an intermediate approach.

In many African countries, PNC provided by TBAs or community health workers may currently be a more realistic and feasible avenue for PNC delivery given the geographic and financial barriers women face to access facility care during the early postnatal period. Furthermore, where formal health systems are overstretched, such health workers may serve as a crucial backstop to SBAs. There is currently a global focus on a linked approach to newborn health which includes a strong referral system between community and facility care [11, 12, 18]. Many countries are also encouraging TBAs to work closely with the formal health sector and provision of PNC follow-up has been shown to be a suitable role [15] as countries work to scale-up the presence of SBAs. These providers should be given training in elements of PNC that they could appropriately administer. Training on when to refer to a skilled provider would also be essential.

Limitations of our study include endogeneity due to omitted variables including birth weight Many women who deliver at home do not know their child’s birth weight, and low birth weight is a strong predictor for neonatal mortality. In addition questions on gestational age, another important predictor variable, are not included in the DHS. A second key limitation is that we can only study associations and not causality due to the cross-sectional nature of the data, though we do know the timing of death and the PNC check-ups. Reverse causality is possible in the association between neonatal death and not breastfeeding whereby newborn illness could result in his/her inability to breastfeed rather than not breastfeeding resulting in death. Thirdly, multiple births could slightly bias the results as these births may be more likely to be attended by a SBA than a singleton birth, but the numbers of multiple births were small. The main limitation is our inability to study associations between PNC and deaths on the first day of life. As mentioned earlier, the DHS records deaths occurring within a few minutes of life and after several hours the same way. These deaths are recorded as occurring on day one so determining which newborns died before they were eligible for PNC was not possible. Given the high numbers of deaths that occur on the very first day of life, we would encourage future large scale surveys to classify deaths on the first day by hours.

Despite the limitation of the study, we were able to provide evidence to support efforts to promote PNC as a means to prevent neonatal deaths in Sub-Saharan Africa. This is an important topic for the global health community as countries are focusing on scaling-up PNC services to reach more newborns and save more lives.

## Conclusion

This study is among the first to evaluate associations between PNC by provider type and neonatal death in Sub-Saharan Africa. Overall findings from this study indicate that countries should continue to promote PNC as a means to prevent neonatal mortality and improve newborn health. Current levels of PNC provision are quite low, and this study provides evidence to support scale-up. As countries in sub-Saharan Africa work to increase both the number of SBAs and the number of women delivering in health facilities, provision of PNC by trained TBAs or community health workers may be used as an intermediate strategy to save the youngest lives. Such workers such be trained on essential components of PNC and the importance of referral for sickness or complications. By using a combined community and facility approach, PNC can reach more newborns and thus save more lives.
